# Patients that maintain their pre-injury level of physical activity 3–5 years after ACL reconstruction are, 18 months after surgery, characterised by higher levels of readiness to return to sport

**DOI:** 10.1007/s00167-022-07230-w

**Published:** 2022-11-19

**Authors:** S. Beischer, E. Hamrin Senorski, R. Thomeé

**Affiliations:** 1 Unit of Physiotherapy, Department of Health and Rehabilitation, Institute of Neuroscience and Physiology, The Sahlgrenska Academy, University of Gothenburg, Box 455, 405 30 Gothenburg, Sweden; 2 Sportrehab Sports Medicine Clinic, Gothenburg, Sweden; 3 Department of Orthopaedics, Sahlgrenska University Hospital, Mölndal, Sweden

**Keywords:** Knee, Physical inactivity, Sports medicine, Rehabilitation

## Abstract

**Purpose:**

To characterise patients who had returned to their pre-injury physical activity (PA) or higher at 18 months and maintained that level of PA 3–5 years after the primary ACL reconstruction and to describe the level, frequency, and type of PA participation during the first 5 years after ACL reconstruction

**Method:**

Data, from follow-ups at 18 months and 3–5 years after an ACL reconstruction, were extracted from a rehabilitation-specific register. Patients, 15–65 years of age, were included. The data comprised patient-reported outcomes and the results from two questions with respect to the level, frequency, and type of PA. Comparisons were made between patients who had and had not maintained their pre-injury level of PA at the follow-up 3–5 years after an ACL reconstruction.

**Results:**

A total of 272 patients met the inclusion criteria. The mean follow-up time was 3.8 years (min–max: 2.9–5.1) after the ACL reconstruction. Of patients who had returned to their pre-injury or a higher level of PA at the 18 month follow-up (*n *= 114), 68% (*n* = 78) maintained that level at the 3- to 5-year follow-up after ACL reconstruction. These patients reported a higher level of psychological readiness to return to sport (98 versus 79; *p *= 0.013). Moreover, these patients were 6.0 years older (*p* = 0.016) and were characterised by male sex (56% versus 44%; *p *= 0.028) and a lower level of pre-injury PA (*p* = 0.013). At the follow-up 3–5 years after the ACL reconstruction, more than 90% met the recommendations for PA. However, the prevalence of physical inactivity had increased and the involvement in organised PA had decreased compared with the 18-month follow-up.

**Conclusions:**

Two out of three patients who have returned to their previous level of PA at 18 months can be expected to maintain that level, 3–5 years following ACL reconstruction. These patients were mainly characterised by a higher level of psychological readiness, especially in patients who participated in knee-strenuous sport and were younger than 20 years of age. The results of this study suggest that patients become more physically inactive over time, implicating the importance of clinicians helping patients find a suitable PA that may help patients maintain an active lifestyle.

## Introduction

Physical activity (PA) is one of the most important factors when it comes to preventing and managing non-communicable diseases, such as cardiovascular diseases, cancer, and diabetes [10, 34]. According to the World Health Organisation’s (WHO) guidelines on PA and sedentary behaviour from 2020, all adults (18–64 years of age) should participate in at least 150–300 min of moderate-intensity aerobic PA weekly; or 75–150 min of vigorous-intensity aerobic PA; or an equivalent combination of moderate- and vigorous-intensity activity [10]. For additional health benefits, adults and adolescents are also recommended to perform “muscle strengthening activities at moderate or greater intensity that involve all the major muscle groups on two or more days a week” [10].

A severe knee injury, such as an injury to the anterior cruciate ligament (ACL), often entails a period of reduced frequency and intensity of PA and a change in the type of PA [15]. Historically, ACL injury research has mainly focused on aspects of returning patients to sport, which has also been used as an outcome of successful treatment [1]. However, only about 50% of patients with an ACL reconstruction return to competitive sport and about two-thirds return to their pre-injury level of sport within the first 7 years after the reconstruction [2, 3]. Patients who succeed in returning to sport after an ACL reconstruction have been characterised by lower levels of fear of re-injury [27], higher self-efficacy of knee function [6, 18], greater motivation to return to sport [4, 5, 13], and higher levels of psychological readiness to return to sport [37]. In the light of self-reported knee function, patients who return to sport reporting less impairment during sport and recreation and higher levels of knee-related quality of life (QoL) [14, 18]. Ithurburn et al. [19] reported no differences in self-reported knee function between patients who had returned to pre-injury sport and had maintained versus not maintained that level of sport 1 year after the return. However, it is not known whether there are differences in psychological factors between these patients.

Regardless of whether or not a patient returns to sport, it is of great importance to maintain any PA to minimise the risks of non-communicable diseases [34]. Specifically, the risk of developing radiographic signs of osteoarthritis (OA) is increased in patients after an ACL reconstruction [17, 25]. Exercises therapy, including strength training of the knee extensors muscles, is recommended as muscular weaknesses of the knee extensors have been associated with an increased risk of developing symptoms of knee osteoarthritis [28]. In patients who have developed OA, exercise therapy is strongly recommended. Therefore, to achieve the recommended levels of PA is a minimum level of PA for patients who develop OA after an ACL reconstruction [26]. Although previous studies [7, 20, 22] have assessed the level of PA from a long-term perspective, a few studies have investigated the frequency of PA in patients after ACL reconstruction. To better understand PA habits, there is a need for a more detailed description of the level, frequency, and type of PA in a time when most patients have completed their rehabilitation after an ACL reconstruction.

The aim of this study was to 1) characterise patients who had returned to their pre-injury PA or higher at 18 months and had maintained that level of PA at a follow-up between 3 and 5 years after the primary ACL reconstruction, and 2) describe the level, frequency, and type of PA participation during the first years after completion of the rehabilitation. The null-hypothesis was that there would be no differences in psychological factors or self-reported knee function between patients who maintained and did not maintain PA 3–5 years after the primary ACL reconstruction.

## Materials and methods

Ethical approval was obtained from the Swedish Ethical Review Authority (registration numbers: 2020–02501) and the study was conducted according to the World Medical Association Declaration of Helsinki. The data were extracted on June 14, 2021.This study, based on prospectively collected data from a rehabilitation-outcome register, Project ACL, was designed following the recommendation of the STROBE statement [33]. Project ACL consists of over 3000 patients with an ACL injury with outcome data from patient-reported outcomes (PROs) and tests of muscle function (strength tests and hop tests). Evaluations are scheduled at predefined follow-ups (10 weeks, 4, 8, 12, 18 months and yearly up to 5 years and every fifth year thereafter), starting with the ACL injury or reconstruction as baseline. All the patients included in the register were given written information about the study and informed consent was obtained. All patients can withdraw from participation at any time, without any further explanation.

### Eligibility criteria

Patients registered in Project ACL with at least an 18-month follow-up after the primary ACL reconstruction were assessed for eligibility. The 18 months follow-up was chosen as it corresponds to a time when most patients have completed their rehabilitation after an ACL reconstruction [24]. The following inclusion criteria were used; a primary ACL reconstruction between January 2013 and September 2017, 15 to 65 years of age at the primary ACL reconstruction and at least one follow-up at 3, 4 or 5 years after the ACL reconstruction. Patients were excluded if they reported a new ACL injury since their last follow-up or another injury or illness (e.g., ankle sprain, post COVID-19 condition, or sciatica) that might affect their ability to be physically active at the 3- to 5-year follow-up.

### Return to physical activity

A modified version of the Tegner Activity Scale (Tegner) [6, 32] was used to assess the level of PA pre-injury, at 18 months and at 3–5 years after the ACL reconstruction. The Tegner is graded from 1 to 10, with 1 representing the least strenuous knee activity and 10 representing the most strenuous knee activity, such as rugby and international football. The Tegner has been reported to have acceptable test–retest reliability, with an ICC of 0.8 for patients with an ACL injury or reconstruction [8]. The modified version does not contain the “0” score, which represents “sick leave or disability pension because of knee problems” in the original version. Furthermore, the modified version of the Tegner has recreational sports activities as a choice up to level 9.

Patients who reported the same or higher Tegner at the 18-month follow-up compared with the pre-injury score were defined as having returned to their pre-injury level of PA. Based on the reported Tegner at the 3- to 5-year follow-up, the included patients were divided into the following groups: 1) same or higher PA level compared with pre-injury level (same or higher) and 2) lower PA level than the pre-injury level (lower). For the primary aim of the study, only eligible patients who had returned to their pre-injury level of PA at 18 months after ACL reconstruction were included. Patients are continuously included in Project ACL regardless of the time that has passed since the ACL injury or reconstruction. As a result, some patients only had Tegner data from the 18-month and the 3-year follow-ups, while other patients had data from all follow-ups. Moreover, in September 2020, the 4-year follow-up was excluded from the standard schedule of follow-ups. For patients who had data from more than one of the follow-ups 3–5 years after the ACL reconstruction, data from the latest follow-up were included for the main analysis.

### Study outcomes

The Knee injury and Osteoarthritis Outcome Score (KOOS) [29, 30] was used to evaluate the patients’ opinions of their knee and associated problems during the previous week. The scale comprises 42 items in five subscales including pain, other symptoms, activities of daily living, function in sport, and recreation and knee-related QoL. Standardised answer options are given, and each question is assigned a score from 0 to 4. Each subscale score is calculated independently, by dividing the mean score for each subscale by 4 and then multiplying the result by 100 (100 indicates no problem and 0 indicates extreme problems). The KOOS has been reported to have acceptable test–retest reliability for patients with a knee injury (ICC = 0.85–0.93) [30]. In Project ACL, the subscale of activities of daily living is only completed preoperatively and at yearly follow-ups. As a result, the subscale of activities of daily living, at the 18-month follow-up, was excluded in this study.

A modified version of the Knee Self-Efficacy Scale (K-SES_18_) was used to assess patients’ knee-related self-efficacy [6]. The K-SES_18_ consists of two subscales, present knee self-efficacy and future knee self-efficacy. Patients rate each item on an 11-point Likert scale, ranging from 0 = not at all certain to 10 = very certain. The mean value of each subscale was used for further analysis. The K-SES_18_ has been reported to have acceptable reliability and validity to assess knee self-efficacy in patients, 16–50 years of age, after an ACL injury or reconstruction [6].

The Swedish version of the ACL Return to Sport after Injury Scale (ACL-RSI) [23, 36] was used to assess psychological readiness to return to sports participation 18 months after ACL reconstruction. The Swedish version consists of 12 items and has been reported to be valid, internally consistent, and reliable after ACL reconstruction (ICC = 0.89) [23]. Patients rate each item on a 10-point Likert scale. The total score for all 12 items on the ACL-RSI was used for further analysis.

Two single questions were used to assess the type of PA/sport pre-injury at 18 months and 3, 4 and 5 years after the ACL reconstruction: 1) Have you participated in any PA/sport during the last month? If “yes”, enter the main PA/sport and 2) At which level have you participated in PA or sport? Question 1 was an open-ended question and Question 2 had the following fixed-alternative responses:

- Physically inactive (less than 30 min of PA/day or less than 150 min/week)
- Physically active (more than 30 min of PA/day or more than 150 min/week)
- Exercising (up to 2 days/week of regular exercising)
- Active exercising (regular exercising 3–7 days/week without regularly participating in competition)
- Active competition (regular exercising 3–7 days/week with regular participation in competition)
- Elite (division 2–3, youth elite or junior elite)
- National elite (highest division)
- International elite

To assess the type of pre-injury PA, the results from a single question, “Enter the physical activity/sport in which you are mainly involved”, were used. This question was answered with free text upon registration in the project. The questions regarding level and type of PA/sport have been developed for Project ACL by the research group. First, the scientific literature with respect to the topic was reviewed. Then, to ensure face validity, three physiotherapists, all with experience of patients with an ACL injury, took part in the development of the questions. Data from the 18-month follow-up and from the latest registered follow-up between 3 and 5 years after the ACL reconstruction were extracted from Project ACL.

## Statistical analysis

Statistical analysis was performed using the IBM SPSS Statistics for Windows, version 25, 2017 (IBM Corp., Armonk, N.Y., USA). The sample-size calculation was made for the main outcome (ACL-RSI at the 18-month follow-up), where the estimated mean was based on data in a previous study [35]. A sample-size calculation showed that a total sample size of 72 patients were required to be able to identify a 13.4-point difference, corresponding to the minimal detectable change (MDC) [35] between groups with 80% power at an alpha level of 0.05.

Descriptive statistics for patient demographics and outcomes were reported with the count and proportion for categorical variables. Continuous variables were reported as the mean or median and standard deviation (SD) or minimum and maximum. The frequency of the 12 most common types of PA was reported at pre-injury, 18 months and 3, 4 and 5 years after ACL reconstruction.

For comparisons between patients with complete data and those lost to follow-up and between patients who maintained and did not maintain PA 3–5 years after the primary ACL reconstruction, the Fisher’s exact test for dichotomous variables was used. Pearson’s chi-square test for ordered categorical variables and the Mann–Whitney U test for non-parametric and non-normally distributed data. To assess normality for the variables of age, height, and weight, the *z*-score for skewness and kurtosis was calculated. As some of the continuous variables were not normally distributed, presented in Table 4 (Appendix) all between-group analyses were performed with non-parametric tests. The data were defined as normally distributed if the z-score was within ± 2.58. Significance tests were conducted at the 5% level.

## Result

**Fig. 1:**
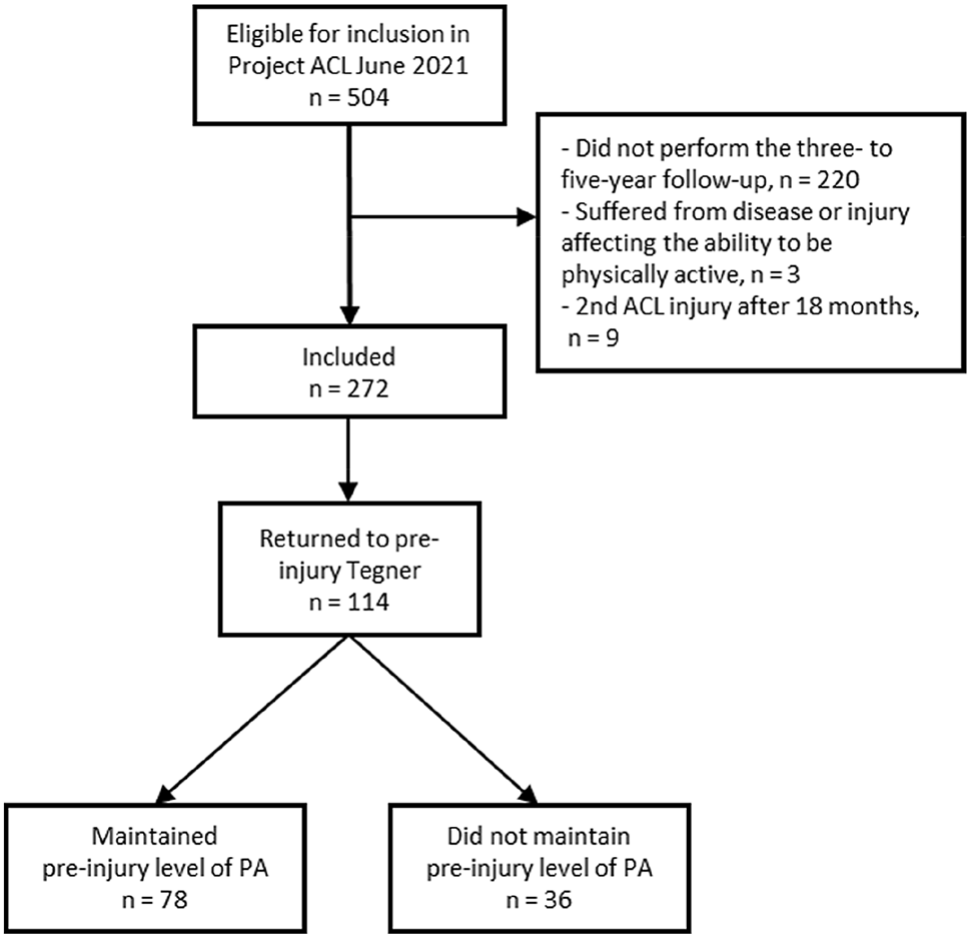
Flowchart of the inclusion process. *PA* physical activity

A total of 272 patients met the inclusion criteria (Fig. 1). Of the eligible patients (*n* = 504), 220 patients had no follow-up 3–5 years after the ACL reconstruction. In addition, three patients were excluded as they reported disease or an injury affecting their ability to be physically active 3–5 years after the ACL reconstruction and nine patients were excluded as they sustained a second ACL injury between the 18-month and 3- to 5-year follow-ups. There were no differences between included patients (*n* = 281) and patients lost to follow-up (*n* = 220), Table 1.

**Table 1:** Baseline demographics, anthropometrics, and drop out analysis

	Lost to follow-up	Included	*P* value
	(*n* = 220)	(*n* = 272)	
Patient sex			
Female	120 (55)	141 (52)	n.s
Time between ACL injury and reconstruction (years)	0.4 (0.0–20.8)	0.4 (0.0–20.6)^a^	n.s
Age (years)^b^	27.4 (16.6–58.7)	28.9 (16.5–65.3)	n.s
BMI	23.8 (18.4–34.0)^a^	23.7(16.7–34.0)^a^	n.s
Tegner pre-injury			n.s
1–5	50 (22.7)	58 (21.3)	
6	28 (12.7)	31 (11.4)	
7	26 (11.8)	56 (20.6)	
8	41 (18.6)	51 (18.8)	
9	52 (23.6)	57 (21.0)	
10	23 (10.5)	19 (7.0)	
Graft			n.s
HT	153 (87.4)	215 (89.6)	
PTBT	19 (10.9)	19 (7.9)	
QT	1 (0.6)	0 (0)	
Allograft	2 (1.1)	6 (2.5)	
Missing (n)	45	32	

For categorical variables *n* (%) is presented. For continuous variables, median (min–max) is presented. For comparison between groups, the Fisher’s exact test was used for dichotomous variables, the Pearson’s Chi-square for nominal variables, and the Mann–Whitney U test for ordered categoric and continuous variables.* BPTP* bone–patella bone–tendon autograft, *HT* Hamstring tendon autograft, *n.s* not significant, *QT* Quadriceps tendon graft, *Tegner* Tegner Activity Scale: ^a^missing value for 2 patients;^b^at the 18 month-follow-up

Figure 1 shows the inclusion of patients and the stratification into different groups for the comparison analysis. Of the included 272 patients, 114 (42%) had returned to their pre-injury level of PA at the 18-month follow-up.

On average, the included patients had a follow-up at 3.8 years (min–max: 2.9–5.1) after their ACL reconstruction. For 70% of the patients (191/272), more than 5 years had passed since the ACL reconstruction. For these patients, the last registered follow-up was extracted from the 3-, 4-, and 5-year follow-ups for 28% (53/191), 26% (50/191), and 46% (88/191), respectively.

In patients who had returned to their pre-injury level of PA (*n* = 114) at 18 months, 68% (*n* = 78) maintained the same level of PA 3–5 years after the ACL reconstruction. Patients who maintained their pre-injury level of PA reported higher levels of psychological readiness to return to sport (median: 98 versus 79; *p* = 0.013) at the 18-month follow-up, were 5.0 years older (*p* = 0.016), had a lower pre-injury Tegner (*p* = 0.005), and comprised more men (56% versus 44%; *p* = 0.028) compared with patients who did not maintain their PA (Tables 2 and 3). Because patients who maintained their pre-injury level of PA comprised a larger proportion of patients with a pre-injury level of PA on Tegner < 6 (42% versus 19%; *p* = 0.005; Table 2), a sub-group analysis of patients with a pre-injury level of PA on Tegner ≥ 6 was carried out. The rationale for this was based on the assumption that maintaining a lower Tegner is less demanding than maintaining a higher level of PA. This sub-group analysis confirmed the findings that patients who had maintained their pre-injury level of PA 3–5 years after ACL reconstruction were characterised by higher levels of psychological readiness to return to sport (102 versus 79; *p*-value = 0.008) at the 18-month follow-up. The sub-groups analysis revealed no other differences between patients who maintained and did not maintain their pre-injury level of PA. Moreover, since patients who maintained their pre-injury level of PA were 5 years older (Table 2), a sub-group analysis stratified by the age-groups 1) younger than 20, 2) 20 < 30 years, and 3) ≥ 30 years of age was carried out. This sub-group analysis revealed that patients younger than 20 years of age who had maintained their pre-injury level of PA 3–5 years after ACL reconstruction were characterised by higher levels of present (9.8 vs 9.4; *p* = 0.019) and future (9.5 versus 8.7; *p* = 0.039) self-efficacy, higher levels of psychological readiness to return to sport (111 versus 78; *p* = 0.011), and enhanced function in sports and recreation (95 versus 80; *p* = 0.023) at the 18-month follow-up. No other differences between patients, younger than 20 years of age, who maintained and had not maintained their pre-injury level of PA were found. For patients 20 < 30 years and ≥ 30 years of age, respectively, no differences between patients who maintained and did not maintain their pre-injury level of PA were found (Table 2).

**Table 2:** Patient demographics in patients who had or had not maintained their pre-injury level of physical activity at the 3- to 5-year follow-ups, in all patients and stratified by age-groups

	All patients		*P* value	Patients < 20 years of age		*P* value	Patients 20 ≥ 30 years of age		*P* value	Patients ≥ 30 years of age		*P* value
	Maintained PA	Not maintained PA		Maintained PA	Not maintained PA		Maintained PA	Not maintained PA		Maintained PA	Not maintained PA	
	*n* = 78	*n* = 36		*n* = 17	*n* = 14		*n* = 27	*n* = 14		*n* = 34	*n* = 8	
Patient sex												
Male	56	36	0.028	29.4	28.6	n.s	29.6	70.4	n.s	58.8	37.5	n.s
Age at 18-month follow-up (years)	28.9 (16.5–60.0)	23.9 (16.5–56.6)	0.016									
BMI (kg/m^2^)	24.2 (19.5–34.0)^a^	23.7 (20.0—27.4)	n.s	23.2 (19.5–28.7.5)	22.9 (20.0–27.4)	n.s	25.0 (19.7–31.5)	24.0 (21.2–26.2)	n.s	24.8 (20.2–34.0)	23.4 (22.5–26.2)	n.s
Tegner pre-injury												
1–5	42.3	19.4	0.005	5.9	–	n.s	37.0	28.6	n.s	64.7	37.5	n.s
6	6.4	8.3		–	–		7.4	7.1		8.8	25.0	
7	17.9	19.4		17.6	7.1		11.1	35.7		23.5	12.5	
8	17.9	25.0		41.2	42.9		22.2	14.3		2.9	12.5	
9	12.8	27.8		23.5	50.0		22.2	14.3		–	12.5	
10	2.6	–		11.8	–		–	–		–	–	

For categorical variables, % is presented. For continuous variables, median (max–min) are presented. For comparisons between groups, Fisher’s exact test was used for dichotomous variables, the Kruskal–Wallis test was used for ordered categorical variables, and the Mann–Whitney U test was used for non-normally distributed continuous variables. *n.s.* not significant, *PA* Physical Activity, *Tegner* Tegner Activity Scale. Missing value for ^a^one patient

**Table 3:** Patient-reported outcomes in patients who had or had not maintained their pre-injury level of physical activity at the 3- to 5-year follow-ups, in all patients and stratified by age-groups

	All patients			*P* value	Patients < 20 years of age		*P* value	Patients 20 ≥ 30 years of age		*P* value	Patients ≥30 years of age		*P* value
	Maintained PA		Not maintained PA		Maintained PA	Not maintained PA		Maintained PA	Not maintained PA		Maintained PA	Not maintained PA	
	*n* = 78		*n* = 36		*n* = 17	*n* = 14		*n* = 27	*n* = 14		*n* = 34	*n* = 8	
K-SES_18_ present	9.4 (0.7–10.0)		9.4 (5.4–10.0)	n.s.	9.8 (8.9–10.0)	9.4 (7.3–9.9)	0.019	9.2 (6–10.0)	9.5 (7–10.0)	n.s.	9.2 (0.7–10.0)	8.4 (5.4–10.0)	n.s.
K-SES_18_ future	8.5 (1.3–10.0)		8.2 (5.0–10.0)	n.s.	9.5 (3.5–10.0)	8.7 (5.5–10.0)	0.039	8.5 (3.5–10.0)	8.2 (1.3–10.0)	n.s.	8.2 (1.3–10.0)	7.1 (5.0–10.0)	n.s.
ACL-RSI	98 (26–120)^a^		79 (21–16)^b^	0.013	111 (70–120)^c^	78 (26–116)^d^	0.011	90 (42–119)^e^	97 (26–120)^f^	n.s.	97 (26–120) ^f^	87 (21–108) ^g^	n.s.
KOOS													
Pain	94 (14–100)		92 (61–100)	n.s.	97 (78–100)	92 (64–100)	n.s.	92 (67–100)	94 (14–100)	n.s.	94 (14–100)	96 (69–100)	n.s.
Symptoms	86 (32–100)		79 (36–100)	n.s.	89 (57–100)	84 (36–100)	n.s.	86 (46–100)	88 (32–100)	n.s.	88 (32–100)	77 (54–96)	n.s.
Sports	85 (0–100)		80 (30–100)	n.s.	95 (65–100)	80 (45–100)	0.022	85 (45–100)	83 (0–100)	n.s.	83 (0–100)	65 (38–100)	n.s.
QoL	75 (12–100)		69 (38–100)	n.s.	81 (56–100)	69 (38–100)	n.s.	75 (25–94)	75 (12–100)	n.s.	75 (12–100)	69 (38–100)	n.s.

For categorical variables, n (%) is presented. For ordinal variables, the median (max–min) is presented. For comparisons between groups, Fisher’s exact test was used for dichotomous variables and the Mann–Whitney U test was used for ordered categorical variables and non-normally distributed continuous variables. *ACL* Anterior Cruciate Ligament, *K-SES*_*18*_ the 18-item version of the Knee Self-Efficacy Scale, *n.s.* not significant, *PA* Physical Activity, *QoL* Quality of life, *SD* standard deviation, *Tegner* Tegner Activity Scale. Missing value for ^a^ 11, ^b^ 9, ^c^ 2, ^d^ 6, ^e^ 4, ^f^ 5, ^g^ 1 patients

### Type and frequency of PA

**Fig. 2:**
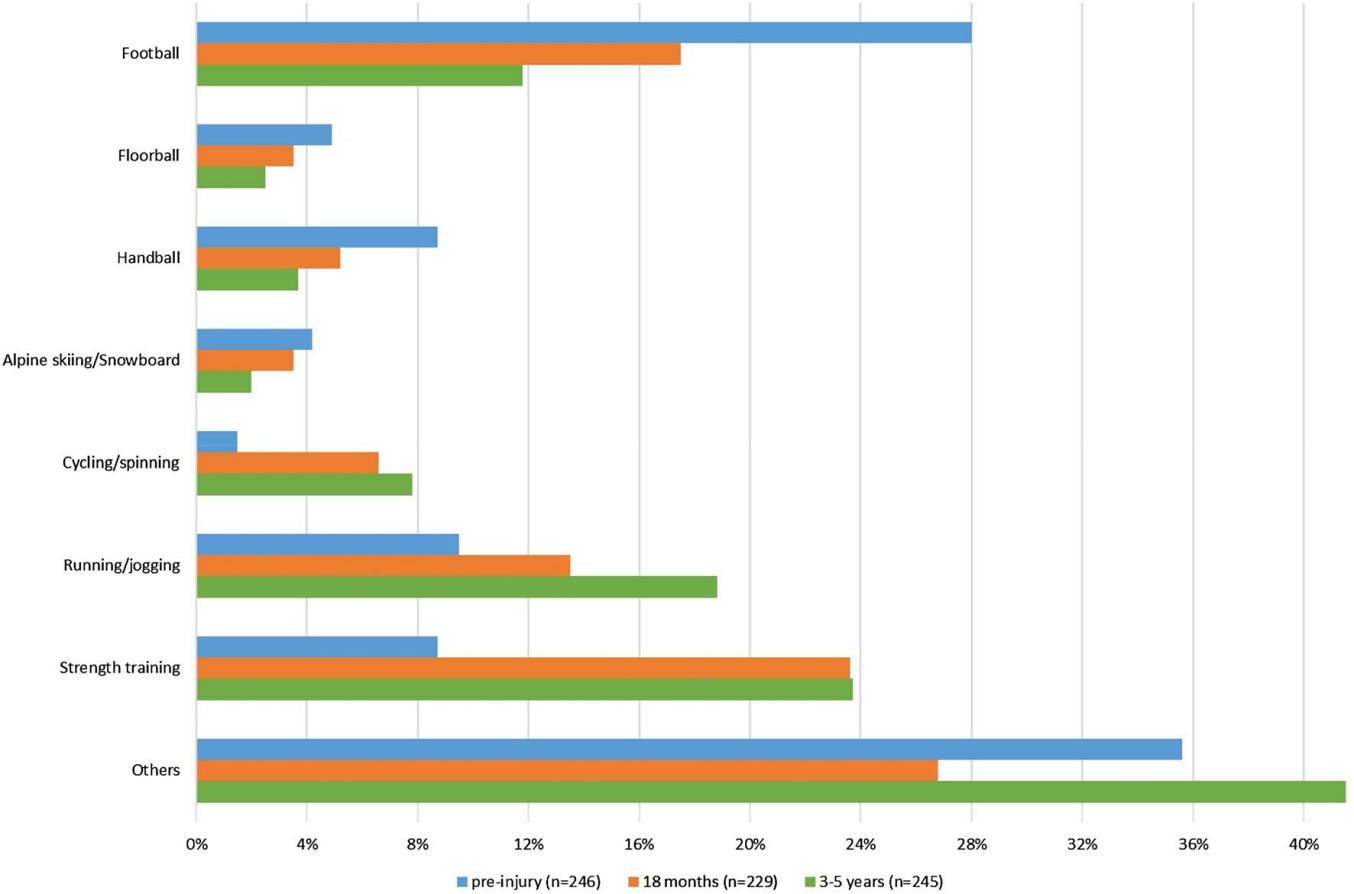
Type of physical activity at 18 months and the follow-up between 3 and 5 years after an ACL reconstruction (corresponding to the latest performed follow-up). Missing values for 8, 43, and 27 patients at the pre-injury, the 18-month and the 3–5-year follow-up, respectively

Figure 2 presents the patients’ main PA before their ACL injury and at 18 months and the follow-up between 3 and 5 years after the ACL reconstruction. Before the ACL injury, the four main PAs were football (28.0%), running/jogging (9.5%), strength training (8.7%), and handball (8.7%). At the following follow-ups, the most common PA was strength training, ranging from 20.2 to 25.7% Participation in organised sports, such as in football, handball, and floorball, decreased from pre-injury to the follow-up between 3 and 5 years for all sports (Fig. 2).

**Fig. 3:**
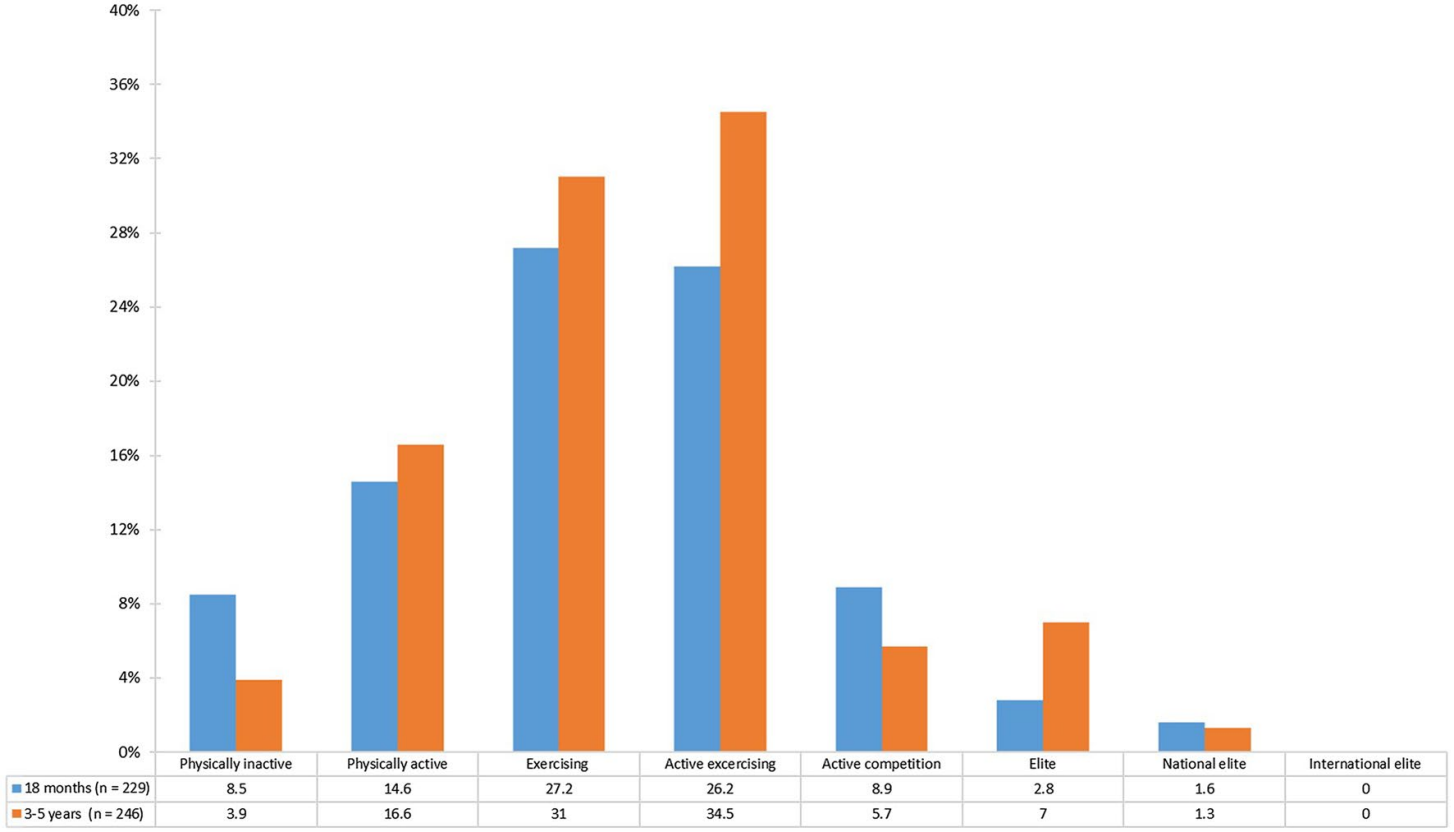
Level of physical activity 18 months to 5 years after an ACL reconstruction. Physically inactive less than 30 min of physical activity/day or less than 150 min/week; physically active more than 30 min of physical activity/day or more than 150 min/week; exercising up to 2 days/week of regular exercising; active exercising regular exercising 3–7 days/week without regular participation in competition; active competition regular exercising 3–7 days/week with regular participation in competition; Elite Division 2–3, youth elite or junior elite; national elite highest division. 3–5 years refers to the latest performed follow-up between 3 and 5 years after ACL reconstruction. Missing values for 43 and 26 patients were present at the 18-month and the 3–5-year follow-ups, respectively

Figure 3 presents the self-reported frequency of PA at 18 months and the follow-up between 3 and 5 years after ACL reconstruction. At the 18-month follow-up, 9/229 patients reported that they were physically inactive according to the WHO. At the same follow-up, 38 patients (16.6%) reported that they were physically active more than 30 min of/day or more than 150 min/week. Three out of these 38 patients were younger than 18 years of age at that follow-up. At the follow-up between 3 and 5 years after the ACL reconstruction, 8.5% of 272 patients reported physical inactivity. At the 18-month follow-up, 14.0% of the included patients reported that they exercised regularly, 3–7 days/week with regular participation in competition, or more, which corresponds to the four highest possible levels of PA (Fig. 3). The corresponding frequency at the follow-up between 3 and 5 years was 13.3.

## Discussion

The main finding in this prospective cohort study was that around two in three patients, who returned to their pre-injury level of PA at 18 months after ACL reconstruction, maintained that level at a follow-up 3–5 years after surgery. Patients who had maintained their pre-injury level of PA were characterised by higher psychological readiness to return to sport, older age, lower pre-injury level of PA and male sex. At the follow-up 3–5 years after the ACL reconstruction, more than 90% met the recommendations for PA. However, the prevalence of physical inactivity had increased from 3.9 to 8.5% and the involvement in organised PA had decreased compared with the 18-month follow-up.

In all, 42% of all included patients (114/272) had returned to their pre-injury level of PA at the 18-month follow-up. Of these, 68% (64/114) had at least maintained the same level of PA 3–5 years after the ACL reconstruction. This means that less than one-third of all the included patients participated at their pre-injury level of PA 3–5 years after their ACL reconstruction, which is lower than the 45% that has previously been reported in patients 2–7 years after ACL reconstruction [3]. Patients who maintained their pre-injury level of PA 3–5 years after the ACL reconstruction were 5 years older than patients who did not maintain their pre-injury level of PA. This finding partly contradicts previous results reported by Ardern et al. [3] who reported that a larger proportion of patients younger than 25 years of age had returned to their pre-injury level of sport 2–7 years after ACL reconstruction compared with patients older than 25 years of age. The discrepancies between the results of the present study and the results reported by Ardern et al. [3] could be explained by the broader inclusion criteria in the present study; patients were included irrespective of their pre-injury type and level of PA, while Ardern et al. [3] included patients who regularly participated in sports. In the present study, patients who had maintained their pre-injury level of sport comprised a higher proportion of patients with a pre-injury Tegner of 1–5 than patients who did not maintain their pre-injury level of sport (42% versus 19%; *p* = 0.005). When only analysing patients with a pre-injury Tegner of ≥ 6, there was no difference in age between the two groups of patients, which confirms previous findings that Tegner is inversely correlated to individuals’ age [9]. Furthermore, the finding that patients who maintained their pre-injury level of PA were characterised by male sex confirms previous literature [3].

The differences in the total score on the ACL-RSI between patients who had returned and maintained their pre-injury level of PA and patients who had returned but had not maintained their pre-injury level of PA, 3–5 years after an ACL reconstruction (98 versus 79, *p* = 0.013), is of interest. In particular, as this finding was confirmed when analysing only the patients with a pre-injury Tegner of ≥ 6 (101.5 versus 79; *p* = 0.008) and in patients younger than 20 years of age (111 vs 78; *p* = 0.011). Psychological readiness to return to sport, as measured by the ACL-RSI, has repeatedly been reported to be associated with a successful return to sport [4, 31, 37] within the first 12 months after ACL reconstruction. It is therefore reasonable to believe that psychological readiness to return to sport is also associated with maintaining PA during the first 5 years after ACL reconstruction, especially in male patients of younger age. However, Type-I errors, i.e., no differences between the groups, could not been ruled out as there were missing values for the ACL-RSI in the two groups who had and had not maintained their pre-injury level of PA. The reason for missing values on the ACL-RSI is that this questionnaire was introduced in Project ACL 1.5 years after the start of the project. Missing values in combination with the small proportion of patients who had returned to their pre-injury level of PA resulted in a limited number of patients in each group, 67 versus 27 patients in the main analysis and only 15 versus 8 in the sub-group analysis stratified by age-groups.

In the light of the increased risk of developing symptomatic knee osteoarthritis after an ACL injury [17, 25], it is of great importance that patients with an ACL injury remain physically active throughout life. In the present study, the included patients appeared to quit organised PA, such as football, handball, and floorball, in favour of strength training and cycling/indoor cycling, when comparing the reported type of PA from pre-injury up to 5 years after the ACL reconstruction. With respect to the recommendation that patients with knee OA should be treated with a combination of aerobic and strength training exercises that load the quadriceps and hamstring muscles at regular intervals [26], it is promising that strength training was by far the most common type of PA at all follow-ups between 18 months and 5 years after the ACL reconstruction. However, the finding that patients after an ACL reconstruction appear to give up organised sports in favour of individual exercising confirm previous findings [16], but is not unique to this group. According to the “Special Eurobarometer 472 data on physical activity” [12], the frequency of exercise and sport is reported to decrease with age. Similarly, younger people are more likely to be engaged in other physical activities, as well as doing vigorous and moderate physical activity [12]. Therefore, the findings of an increased prevalence of physical inactivity and a reduction of involvement in organised PA compared with the 18-month follow-up in the present study may be explained be this age phenomenon. However, this is one of the first study that in detail describe the frequency, level, and type of physical activity in a group of patients after a primary ACL reconstruction. To better understand how patients after an ACL reconstruction differ from healthy individuals, future studies should include a healthy reference group.

Previous studies have reported that ACL-reconstructed patients are less physically active compared with healthy matched controls [7, 22]. In Sweden, 34% of individuals between 16 and 64 years of age do not meet the WHO’s recommendation of 150–300 min of PA per week [10]. Clearly, the proportions of physical inactivity in the present study were lower, ranging from 4% at the 18 month follow-up to about 8%. From the perspective of preventing non-communicable diseases, this result appears promising. However, as these patients, at group level, had a high pre-injury level of PA, reflected by the Tegner, as well as the fact that the majority of patients stated that they were involved in some sort of organised PA before their injury, the proportion of physically inactive individuals must be regarded as too high. To better understand the PA habits of patients after ACL reconstruction and compare the proportions of patients involved in different types and frequencies of PA, a prospective cohort study with a sufficient number of patients using PROs in combination with device-based assessment approaches appears to be necessary [15, 21].

This study has some limitations that were taken into account before conclusions were drawn. First, a limited, yet homogeneous group of patients were included, as strict inclusion criteria were used. The fact that only a minority of patients had returned to their pre-injury PA level at 18 months after their ACL reconstruction resulted, however, in a limited group of included patients. Nevertheless, the results can be regard as generalisable to patients after ACL reconstruction at the age of 15–65 years, as no differences were seen with respect to patient demographics or anthropometrics at the 18-month follow-up between included patients and patients lost to follow-up. Second, the use of PROs and/or single questions to assess PA is associated with limitations [11, 21]. As the Tegner only reflects how knee strenuous the PA is, we combined the Tegner with a single question where patients were asked to enter the main PA in which they had participated during the last month. To assess the frequency of PA, another single question was used, developed, and tested for face validity by the research group. However, as for all subjective measurements of the frequency of PA, it is likely that the question regarding the frequency of PA is also associated with overestimation [11, 21]. Finally, no data with respect to frequency, type, and severity of possibly associated injuries were available. Therefore, identifying patients with associated injuries that may have influenced the patients’ postoperative PA was not possible.

Few previous studies have investigated the characteristics of patients who return to their previous level of PA and maintain at least the same level of PA in the medium-term perspective after an ACL reconstruction. Moreover, this study adds a description of the level, frequency, and type of PA participation during the first 5 years after surgery in a large cohort of patients.

## Conclusion

Two out of three patients who have returned to their previous level of PA at 18 months can be expected to maintain that level, 3–5 years following ACL reconstruction. Patients who maintained their level of PA were mainly characterised by a higher level of psychological readiness, especially in patients who participated in knee-strenuous sport and were younger than 20 years of age. Regardless of what level of PA patients return to, 9 of 10 patients can be expected to meet the recommendations for PA and about 1 out of 3 patients can be expected to exercise regularly, three-to-seven times per week, mainly involved in strength training and running/jogging. However, the results of this study suggest that patients become more physically inactive over time, implicating the importance of clinicians helping patients find a suitable PA that may help patients maintain an active lifestyle.

## Data Availability

The dataset used and/or analysed is available from the corresponding author on request.

## Appendix

**Table 4:** Z-score for skewness and kurtosis for patient demographics at the 18-month follow-up

		Lost to follow-up (*n* = 220)			Included (*n* = 281)		
		Age	Length	Weight^a^	Age	Length	Weight^a^
Skewness		0.782	0.158	0.597	0.747	0.138	0.448
Std. error of skewness		0.164	0.164	0.165	0.145	0.145	0.146
Z-score skewness		4.766	0.962	3.620	5.139	0.952	3.069
Kurtosis		– 0.048	– 0.653	7.740	– 0.352	– 0.730	– 0.048
Std. error of kurtosis		– 0.339	– 0.408	0.654	0.290	0.290	0.291
Z-score kurtosis		0.327	0.327	0.328	– 1.214	– 2.519	– 0.167
